# Liability of Dentists

**Published:** 1883-01

**Authors:** 


### EDITORIAL, ETC.

---

*Liability of Dentists.*—From the New York Press we learn that in a recent case before the Marine Court of that City, a patient having brought suit against Mr. Colton, of Nitrous Oxide Gas fame, to recover damages for injuries alleged to have been caused by a portion of a tooth, which was being extracted, falling down the plaintiff's throat while under the influence of the gas, the Court (Judge McAdams) decided that while a patient was under the influence of an anasthetic which deprived him of the use of his faculties, it was the duty of the dental operator to exercise the highest professional skill and care to avoid every possible danger; and that the circumstances of the case in question were sufficient to show negligence. The jury accordingly found a verdict against Mr. Colton and awarded the plaintiff five hundred dollars damages.

In this case it was alleged that a portion of the tooth escaped from the forceps, and passing into the throat, for four weeks occasioned a cough and irritation until it was finally expectorated.

This decision is of importance to dentists, as frequently law-suits are resorted to by patients who imagine themselves sufferers by unavoidable accidents, and where juries award damages on account of proneness to condemn through ignorance or inability to judge correctly of the merits of such cases.

